# Effects of lyophilization and storage temperature on *Wuchereria bancrofti* antigen sensitivity and stability

**DOI:** 10.1186/s13104-018-3586-0

**Published:** 2018-07-11

**Authors:** Edem Y. Agbozo, Edward Dumashie, Daniel A. Boakye, Dziedzom K. de Souza

**Affiliations:** 1 Accra Technical University, Accra, Ghana; 2 Department of Parasitology, Noguchi Memorial Institute for Medical Research, University of Ghana, Accra, Ghana

**Keywords:** *Wuchereria bancrofti* antigen, Lyophilization, Dried blood spots, Freeze-drying

## Abstract

**Objective:**

Antigen-based rapid diagnostic tests for Lymphatic filariasis (LF) do not come with external quality control (QC) materials, and research and disease control programmes rely on stored positive samples. This study was undertaken to evaluate the use of lyophilized *Wuchereria bancrofti* antigen positive plasma samples to serve as QC materials for LF diagnostic tests. 10 well characterized *W. bancrofti* positive samples were lyophilized and stored at 4, 28 and 40 °C. The samples were evaluated using the Alere Filariasis Test Strips before lyophilization, and after 1 and 3 months of storage. The sensitivity and stability of the lyophilized samples were evaluated.

**Results:**

The results revealed a loss of sensitivity and stability with increasing temperature and duration of storage. The results are further discussed in terms of the use of dried blood spot (DBS) in diagnostic studies on LF, and the need for thoughtful DBS preparation and storage.

## Introduction

Lymphatic filariasis (LF) is found in tropical and sub-tropical regions of the world, where it is a major public health problem [1]. The availability of tools and strategies for the control of the disease [2, 3] led to World Health Assembly resolution (WHA 50.29) calling on member states to work towards the elimination of LF as a public health problem by 2020 [4]. The World Health Organization (WHO) launched the Global Programme to Eliminate Lymphatic Filariasis (GPELF) in 2000, with the principal objective of breaking the cycles of transmission of *Wuchereria bancrofti* and *Brugia* spp. through the application of annual mass drug administration (MDA) [5] to entire at-risk populations for a period of 5–7 years. Thus, more than 6.7 billion cumulative treatments were provided to disease endemic communities by 2015 [6]. Impact evaluation assessments are based on the prevalence of infection using *W. bancrofti* antigen rapid diagnostic tests (RDTs) or microscopy for microfilaria presence [7].

As part of the GPELF, millions of RDTs as either the BinaxNOW immunochromatographic (ICT) cards [8] and the Alere Filariasis Test Strips (FTS) [9] have been and are currently being used in LF endemic countries, being integrated in LF endemicity mapping, MDA monitoring and stopping, and post-MDA surveillance [7, 10]. The BinaxNOW ICT and Alere FTS detect circulating filarial antigen (CFA)—a 200 kDa *W. bancrofti* antigen—in human blood [9]. Though the BinaxNOW ICT was the first to be developed, challenges with its short shelf life of 3 months at ambient temperature, cost, narrow time window for reading test results and false-positive rates prompted the need for the development of the improved Alere FTS [9]. The Alere FTS is an in vitro, visually read, immunochromatographic test for the qualitative assessment of *W. bancrofti* antigen in blood, utilizing a colloidal gold-labeled polyclonal antibody (PAb) and a monoclonal antibody (MAb) specific for *W. bancrofti*. In a positive sample, *W. bancrofti* antigen combines with the gold-labeled PAb, which is then captured by the MAb forming a pink diagnostic line. While the Alere FTS has an in-built internal procedural control, the use of external controls is recommended to ensure the test reagents are working and that the test is correctly performed. Research and LF control programmes therefore rely on stored samples as quality control (QC) materials. These samples are stored either in the form of plasma or serum in fridges/freezers, or dried blood spots (DBS) stored on silica gel and/or in fridges/freezers. This study sought to test the use of lyophilized (freeze-dried) *W. bancrofti* antigen positive plasma, adapted from a similar method used for HIV rapid tests [11] and malaria [12], as QC material for Alere FTS. Lyophilization was used because it provides a better temperature and humidity-control during the drying process, compared to DBS [13, 14].

The samples used for this study are blood samples collected from LF positive individuals, as part of ongoing research activities [15]. Briefly, study participants were tested during the day for *W. banrofti* antigen using the Alere FTS. Individuals positive for antigen were followed for night blood collection, for the identification of microfilaria. From each participant, 2 ml of night blood was collected (in EDTA coated tubes). 1 ml of blood was analyzed for microfilaria using nucleopore filtration. The remaining blood samples were centrifuged, the plasma separated from the pellets, and frozen at − 80 °C.

## Main text

### Methods

#### Dried *W. bancrofti* infected blood preparation

For baseline reactivity (6 months after storage), 10 frozen plasma samples with known parasite counts (Table 1) were thawed at room temperature and tested on the Alere FTS following the manufacturer’s instructions. From the remaining plasma samples, six (6) aliquots (75 µl each) per sample were lyophilized using a freeze drier (Lyotrap, Ultra Freeze Dryer LF/LYO/04/1, LTE Scientific). Two aliquots from each of the samples were stored in a refrigerator set at 4 °C, at room temperature (28 °C ± 3 °C) and in a dry incubator set at 40 °C, respectively. These were tested after a period of one and 3 months.

**Table 1:** Results of Alere FTS testing at baseline, one and 3 months storage at different temperatures

Samples	Microfilaria/ml of blood	Baseline testing	Results at 1 month			Results at 3 months		
			4 °C	28 °C	40 °C	4 °C	28 °C	40 °C
A	1	+++	+++	+	+++	+++	+	++
B	1	+++	+++	++	++	+	−	+
C	6	+	+	+	+	+	+	+
D	43	++	+	+	+	+	+	−
E	7	+++	++	++	++	++	−	+
F	1	+++	++	++	+	++	+	+
G	6	+++	++	++	++	+	−	−
H	1	++	−	−	−	−	−	−
I	57	+++	+++	+++	++	++	+	+
J	2	++	+++	+++	+++	+	−	−
Number positive		10	9	9	9	9	5	6
Sensitivity		100%	90%	90%	90%	90%	50%	60%
Mean intensity score		2.5	2.0	1.7	1.7	1.4	0.5	0.7
Standard deviation		0.707	1.054	0.949	0.949	0.843	0.527	0.675
Standard error of mean		0.224	0.333	0.300	0.300	0.267	0.167	0.213
p values			0.188	0.055	0.055	0.008	0.004	0.004

p values represent the comparison of means to the baseline

#### Dried blood rehydration and FTS testing

On the day of testing (1 and 3 months after storage), the lyophilized samples were rehydrated with 1 × PBS solution (pH 7.3). 75 μl of PBS was added to the sample tube, and incubated at room temperature for 1 h. The mixture was gently mixed using a pipette and transferred onto the Alere FTS for reactivity testing.

#### Reading and scoring of test results

For all tests undertaken (i.e. baseline, 1 and 3 months) the results were scored as negative (test band absent = 0) or positive (test band present). Even though the Alere FTS is a qualitative tool, the intensity of the test line was assessed visually to provide a semi-quantitative score as previously described [16]. Thus, positive results were scored on a scale of 1–3; 1 (+) being a test band being lighter in intensity than the control band, 2 (++) being a test band having a similar intensity as the control band, and 3 (+++) being a test band with a brighter intensity than the control band. The control band has to be positive for any test to be valid. A false negative test was defined as a sample identified as positive at baseline (before lyophilization) and appearing as negative after lyophilization. All readings were undertaken and scored by two individuals.

#### Data analysis

The baseline test results before lyophilization were used as the reference standard, in estimating the sensitivity of using the lyophilized samples. The sensitivity was estimated by combining the results for each temperature storage condition and expressed as:

$${\text{Sensitivity}}\; = \;\frac{\text{Total samples positive under each storage condition}}{\text{Total samples positive at baseline}}\; \times \; 1 0 0$$

From the semi-quantitative scoring, the mean intensity scores for the baseline, storage temperature and period of storage were computed and test of significance between results were undertaken using the Wilcoxon matched pairs rank test at 95% confidence interval. Graphs and statistical analyses were undertaken using GraphPad Prism version 7.0 (GraphPad Software, Inc., La Jolla, CA).

### Results

#### Sensitivity of *W. bancrofti* lyophilized samples

The results of the evaluations are presented in Table 1. One sample lost total reactivity after lyophilization, while five others were false negative at 28 and 40 °C after 3 months. From the results, 90% sensitivity was observed when the lyophilized samples were tested after 1 month. After 3 months of storage, the sensitivity of the lyophilized samples stored at 4 °C remained unchanged, while that of the samples stored at 28 and 40 °C decreased to 50 and 60% respectively.

#### Assessing *W. bancrofti* antigen stability

**Fig. 1:**
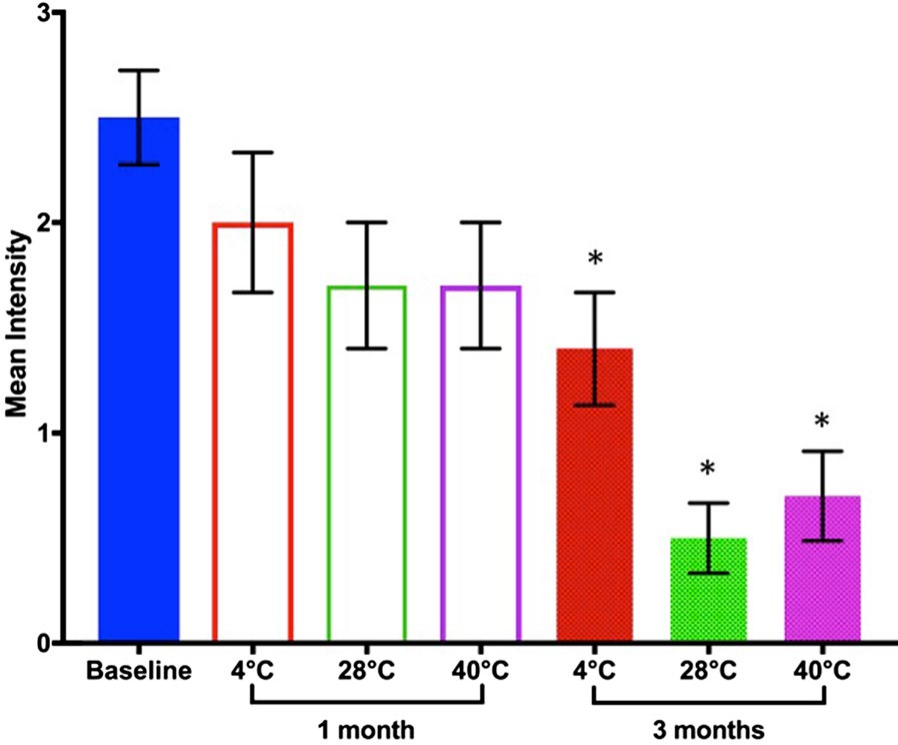
Change in antigen stability with temperature and storage time. Error bars represent the standard errors of the mean. * denotes significance compared to baseline (p < 0.01)

The results showed a decrease in antigen stability with increasing temperature and storage duration (Fig. 1). Compared to the baseline, the results indicate a slight but insignificant loss (p = 0.188) in stability in the lyophilized samples stored at 4 °C for 1 month. However, storage at 4 °C for 3 months resulted in further significant loss in stability (p = 0.008) compared to the baseline, and no differences compared to the 1 month storage (p = 0.125).

Samples stored at 28 °C for a month showed an insignificant loss (p = 0.055) in stability compared to the baseline. Storage at 3 months resulted in a much significant loss compared to the baseline (p = 0.004) and the 1-month storage (p = 0.031).

Storing samples at 40 °C revealed an insignificant loss in stability (p = 0.055) at 1 month, compared to the baseline results. However, storage at 3 months led to a significant loss in stability compared to the baseline (p = 0.004) and the 1-month storage (p = 0.016).

The results indicate that higher temperatures affect the stability of the *W. bancrofti* antigen. Further, long-term storage at higher temperatures results in further loss in antigen stability, with implications for quantitative experiments relying on the use of DBS. Studies evaluating the relative performance of plasma and DBS from *W. bancrofti* positive blood samples showed lower positivity and sensitivity for DBS compared to plasma [17], even though both plasma and DBS were stored at the same conditions of − 20 °C (short term) and − 80 °C (long term). In their study, the only difference in temperature conditions was during the drying stage of the DBS and storage of DBS (in sealed plastic containers) at 4 °C or in hand luggage during transportation. Their studies also showed a higher mean antigen concentration in plasma compared to DBS. Other studies also showed lower positivity and correlations for DBS compared to serum [18, 19], though the storage conditions of the DBS in these studies was not mentioned. Of course, the results from these studies could be explained by the fact that less serum (and therefore antigen) is available in the DBS. However, the effects of temperature on further reducing the stability of the antigen in the DBS, as a result of denaturation, must not be ignored.

Our study therefore provides evidence of the effects of drying and temperature on *W. bancrofti* antigen stability. Studies have shown the effect of temperature on lysosomal enzyme activity during the preparation and storage of DBS [20]. In HIV RDT testing for example, DBS stored at 37 and 45 °C were shown to have good stability until 8 weeks, but when stored at 50 °C it showed good stability until week four. The study concluded that in areas with high temperatures DBS can be stored at room temperature and tested within 4 weeks [21]. In many cases DBS are further stored with desiccants, leading to a further 5% moisture loss during storage [22].

Proteins offer opportunities in disease treatment and diagnostics. However, in order to maintain their properties, proteins need to be stabilized against physical and chemical degradation [23]. Lyophilization improves protein-storage stability, ease of shipping and transportation by removing water [24]. While it has the advantages of; being a low-temperature process with less thermo-denaturation risk, controlling moisture level and enabling better stability of proteins, it also has the disadvantage of inducing conformational instability as a result of the freezing and drying process [13, 14]. Important characteristics of lyophilized products include long-term stability, short reconstitution time and maintenance of the characteristics of the original products. However, lyophilization is not a process that can easily be carried out in the field, unlike the DBS. It requires venous blood collection (compared to DBS from finger-prick blood), cold storage, a lyophilizer, and as such is more time consuming and expensive. Nonetheless for QC purposes, we consider it superior to storage through DBS, during which, it is challenging to control the moisture content coupled with varying environmental (drying) temperatures—under which LF studies are undertaken—which may result in denaturation and loss of protein/antigen activity. In this study, temperatures of 28 °C (± 3 °C) and 40 °C were used as these represent the range of temperatures in LF endemic regions in Ghana and possible temperatures under which DBS may be stored in the absence of a fridge/freezer.

Currently, the Alere FTS kits are not supplied with positive control materials. Until *W. bancrofti* recombinant positive control antigens become commercially available, research and national control programmes will continue to rely on stored samples (where available) as QC materials. Given the progress made through GPELF and the low/limited numbers of positive individuals being detected, sample storage methods should carefully be considered if these are to serve as QC for future studies and programme evaluation activities, most especially for studies relying on quantitative assessment methods and the determination of positive cut-off thresholds. While the use of dried parasitized blood has been evaluated as QC materials for HIV [11] and malaria [12] tests, and may be applicable to LF, further developments of the method aimed at stabilizing the antigen [25], followed by field testing may enhance the utility of lyophilized *W. bancrofti* antigen-positive blood samples in the future.

In conclusion, the results from this study would suggest that dried *W. bancrofti* antigen-positive blood samples stored for long periods above 4 °C should carefully be used in quantitative experiments, given the loss in sensitivity over time. While the DBS is a simpler and cheaper method than collecting samples in EDTA tubes, care must be taken in the storage of these samples in order to obtain the best possible results from their use.

## Limitations

The main challenge to this study was the mall number of samples evaluated. This is due to challenges in obtaining large numbers of well-characterized samples at the current stage of the GPELF in Ghana. While other samples positive with *W. bancrofti* day blood antigen are available, sample size was limited to 10 in order to avoid using all samples at the expense for future positive control materials. The sample volumes available also prevented the preparation of DBS for comparison. Another challenge to the FTS reading may be the subjectivity of the readings by the testing personnel, especially for very faint test bands. However, having two personnel assess the results helped resolve this challenge.

## Abbreviations

DBS: dry blood spot
EDTA: ethylene diamine tetraacetic acid
FTS: filaria test strip
GPELF: Global Programme for the Elimination of Lymphatic Filariasis
HIV: human immunodeficiency virus
LF: lymphatic filariasis
MDA: mass drug administration
NMIMR: Noguchi Memorial Institute for Medical Research
PBS: phosphate buffer saline
QC: quality control
RDT: rapid diagnostic test
WHO: World Health Organization
